# Traffic Sign Recognition Based on the YOLOv3 Algorithm

**DOI:** 10.3390/s22239345

**Published:** 2022-12-01

**Authors:** Chunpeng Gong, Aijuan Li, Yumin Song, Ning Xu, Weikai He

**Affiliations:** 1 School of Automotive Engineering, Shandong Jiaotong University, Jinan 250357, China; 2 Field Centre, Shandong Provincial Agricultural Machinery Scientific Research Institute, Jinan 252100, China; 3 School of Aeronautics, Shandong Jiaotong University, Jinan 250357, China

**Keywords:** traffic sign recognition, YOLOv3, spatial pyramidal pooling structure

## Abstract

Traffic sign detection is an essential component of an intelligent transportation system, since it provides critical road traffic data for vehicle decision-making and control. To solve the challenges of small traffic signs, inconspicuous characteristics, and low detection accuracy, a traffic sign recognition method based on improved (You Only Look Once v3) YOLOv3 is proposed. The spatial pyramid pooling structure is fused into the YOLOv3 network structure to achieve the fusion of local features and global features, and the fourth feature prediction scale of 152 × 152 size is introduced to make full use of the shallow features in the network to predict small targets. Furthermore, the bounding box regression is more stable when the distance-IoU (DIoU) loss is used, which takes into account the distance between the target and anchor, the overlap rate, and the scale. The Tsinghua–Tencent 100K (TT100K) traffic sign dataset’s 12 anchors are recalculated using the K-means clustering algorithm, while the dataset is balanced and expanded to address the problem of an uneven number of target classes in the TT100K dataset. The algorithm is compared to YOLOv3 and other commonly used target detection algorithms, and the results show that the improved YOLOv3 algorithm achieves a mean average precision (mAP) of 77.3%, which is 8.4% higher than YOLOv3, especially in small target detection, where the mAP is improved by 10.5%, greatly improving the accuracy of the detection network while keeping the real-time performance as high as possible. The detection network’s accuracy is substantially enhanced while keeping the network’s real-time performance as high as possible.

## 1. Introduction

Currently, automated driving and intelligent transportation systems (ITS) are the principal applications for traffic sign detection and identification technologies. It can give drivers and autonomous vehicles crucial traffic information so that the latter can make judgments in accordance with the regulations of the road or alert and direct drivers’ operation behaviors in time to reduce traffic accidents. Traffic signs can be broadly divided into three categories: directional signs, warning signs, and prohibition signs. These signs are round or triangular in design, and they are red, yellow, and blue in color. Therefore, classic traffic sign recognition typically uses machine learning techniques to recognize traffic signs or extracts information such as color and shape from traffic signs.

Color segmentation to extract characteristics before classification identification is used in color-based traffic sign detection, which is easily affected by lighting variations. Color segmentation is not influenced by brightness variations, according to a previous literature [1], and uses HIS space to examine only hue and saturation. Due to the high demands of color recognition on variables such as weather and detection distance, the detection approach based on color features can be employed for high-definition image recognition but not for grayscale image recognition [2]. A shape-based traffic sign identification approach on grayscale images was proposed in another literature [3], which transforms triangle traffic sign detection into simple line segment detection, which can properly recognize traffic signs and is unaffected by distance. A support vector machine-based traffic sign detection and recognition system was proposed in another literature [4], which uses the generalization property of a linear support vector machine to first segment the color of traffic signs and then classify the form. The method of detecting color and shape features separately first performs color segmentation to obtain the region of interest, and if the region of interest is not detected, the shape-based detection is no longer performed; second, color segmentation requires a fixed threshold to be set manually, making traffic sign detection complicated and time-consuming. To solve these issues and increase detection performance, one study [5] used the AdaBoost framework to perform simultaneous color and shape modeling detection.

Changes in external conditions, such as light, traffic sign color changes, and so on, can affect color- and shape-based traffic sign detection. The detection impact is unstable, impairing the traffic sign recognition system’s performance and making it vulnerable to traffic sign leakage and false detection. Neural networks are being used more frequently to detect targets as deep learning technology advances; examples of these algorithms include Faster R-CNN [6], SSD [7], and YOLO [8], etc., which are primarily separated into single-stage and two-stage detection approaches. A previous study [9] presented an enhanced detection network based on YOLOv1 to address the issues of low accuracy and slow detection speed of standard traffic sign detection methods. This network enhanced traffic sign detection speed and lowered the hardware requirements of the detection system. Another study [10] suggested a traffic sign detection approach based on enhanced Faster-RCNN, with a 12.1% improvement in mAP, which successfully addressed issues such as low recognition efficiency and raised the precision of traffic sign detection and recognition. In [11], the CCTSDB dataset was obtained by expanding the Chinese Traffic Sign Dataset (CTSD) and updating the marker information based on the improved YOLOv2 target detection algorithm. The CCTSDB dataset only contained three categories of traffic signs, which is insufficient to complete the challenging task of traffic sign recognition. The TT100K [12] dataset, created by Tsinghua University and Tencent in collaboration, was extracted from the Chinese Street View panorama and covers a wide range of lighting and weather conditions, making it more representative of the actual driving environment. Study [13] used DenseNet instead of ResNet in the backbone network of YOLOv3 and experimentally validated it on the TT100K dataset. The algorithm improves the real-time performance of the detection model, but the accuracy and recall tend to be low when it comes to small targets such as traffic signs, which implies serious misdetection. The detection task frequently gets more challenging in target detection tasks, since the target to be detected is typically large, and its features can be easily extracted. Due to the FPN structure that YOLOv3 introduces, it is now able to detect targets at various scales by utilizing multi-scale feature fusion, which is appropriate for complicated traffic scenes and has shown some promise in the detection of small targets. However, there is still some room for improvement for the high-resolution images of the TT100K traffic sign dataset.

In conclusion, the neural network-based approach can successfully address issues with low recognition efficiency, missed detection, and false detection while also enhancing the precision of traffic sign detection and recognition. Neural network-based methods have better accuracy or faster detection than traditional methods but cannot obtain both detection speed and detection accuracy. In addition, most traffic sign detection uses the German Traffic Sign Dataset (GTSDB), and traffic signs in Germany are different from those in China; there are fewer studies on traffic sign detection and recognition in China. Therefore, to address the problems in the above methods, this paper uses the TT100K dataset to train and detect Chinese traffic signs and improve and adjust the YOLOv3 network, mainly with the following improvements:

(1) Add a fourth feature prediction scale of 152 × 152 size to the YOLOv3 network structure to take full advantage of the shallow features in the network to anticipate small targets. To achieve the fusing of local and global features, the spatial pyramid pooling structure is fused.
(2) The distance between target and anchor, overlap rate, and scale are all taken into account when using DIoU loss for faster convergence and more consistent target frame regression. This makes the target frame regression more stable.
(3) The majority of the traffic signs in the TT100K dataset are small- and medium-sized targets, with only a few large targets. As a result, using the original anchor is not a viable option. The K-means clustering algorithm is used to recalculate 12 anchors for the TT100K dataset, and the data augmentation strategy is used to balance and increase the dataset’s imbalanced number of target categories.

## 2. Algorithm Fundamentals

### 2.1. The YOLOv3 Algorithm

**Figure 1:**
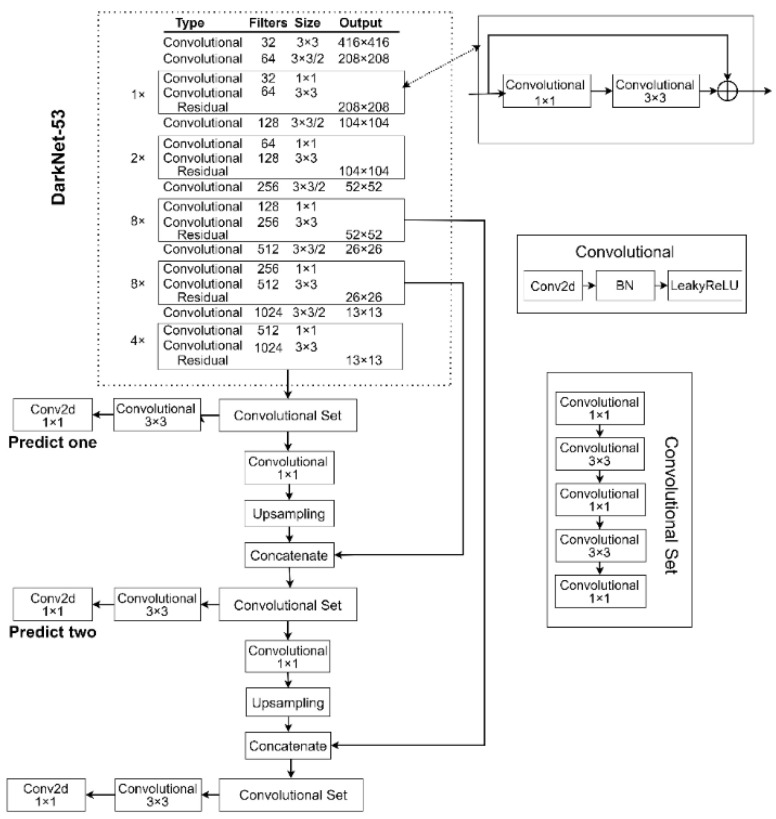
The network structure of YOLOv3.

YOLOv3 [14] is Redmon’s improved, single-stage target detection algorithm based on YOLOv2, which has improved detection accuracy and real-time performance, and outperforms other algorithms in terms of speed and accuracy. YOLOv3 is currently the most popular algorithm in the YOLO family and is widely used in real detection scenarios [15]; the YOLOv3 network structure is shown in Figure 1.

The complete convolutional structure used by YOLOv3 is not constrained by the size of the image input. The pooling and fully connected layers are removed from the entire network structure, and a convolutional layer with a step size of 2 is used instead of the pooling layer for the downsampling operation, which prevents the loss of target information during pooling and facilitates the detection of small targets [16]. In addition, YOLOv3 replaces the DarkNet-19 network structure of YOLOv2 with the DarkNet-53 feature extraction layer.

**Figure 2:**
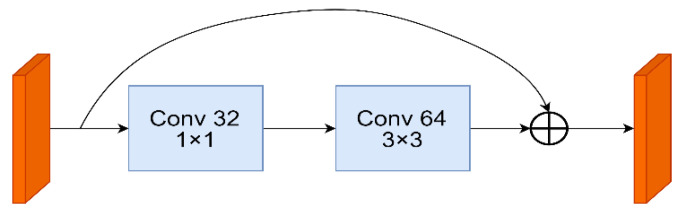
The structure of the residual network.

The DarkNet-53 network, which successfully resolves the gradient problem of the deep network and the loss of original information during the multi-layer convolutional operation to better extract features and improve detection and classification [17], borrows the residual network structure of ResNet [18] and uses the original output of the previous layer as part of the input in the latter layer of the network. As shown in Figure 2, the residual module in YOLOv3 consists of two convolutional layers and a shortcut layer.

**Figure 3:**
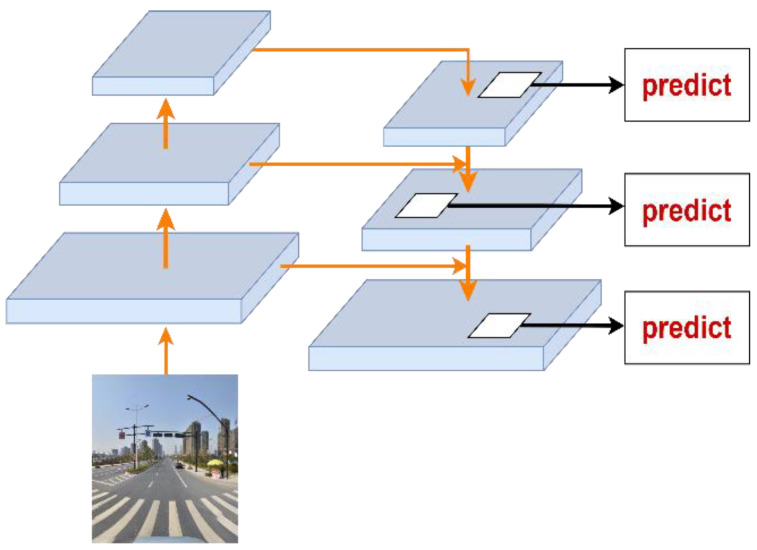
The network structure of FPN.

Furthermore, YOLOv3 uses the notion of a feature pyramid network (FPN) [19] and introduces the feature pyramid network to forecast feature maps at three scales, with detection scales of 13 × 13, 26 × 26, and 52 × 52. The method of feature extraction by the convolutional neural network is bottom-up in the FPN network, and the process of upsampling the convolutional layer feature maps is top-down, as shown in Figure 3.

Deep convolutional layers with wide sensory fields are appropriate for predicting large targets, whereas shallow convolutional layers with small sensory fields are suitable for predicting small targets. The properties of the two layers are combined by lateral connection. As a result, YOLOv3 is capable of predicting objects of varying sizes and is ideal for a variety of sophisticated application scenarios.

### 2.2. Spatial Pyramidal Pooling Structure

**Figure 4:**
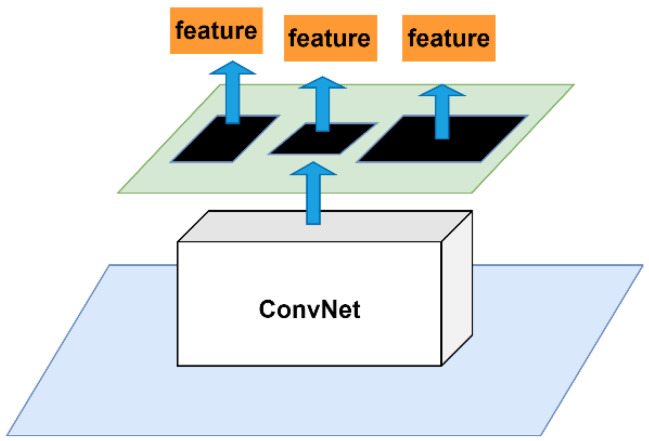
Structure of the SPP module.

The spatial pyramid pooling (SPP) structure [20] solves the problem of repeated extraction of image features by convolutional neural networks and greatly improves the detection efficiency; the SPPNet network structure is shown in Figure 4. To ensure that the resolution of the input image matches the feature dimension of the fully connected layer in a neural network with a fully connected layer, region cropping and scaling operations on the input image are required. Scaling and cropping processes will result in the loss of picture feature information, lowering detection accuracy and affecting detection outcomes; however, scaling and cropping processes will result in the loss of picture feature information, lowering detection accuracy and affecting detection results, whereas SPPNet can overcome the limitation of the fixed size of the input image, saving the computational cost [21].

## 3. Improved YOLOv3

### 3.1. Improved YOLOv3 Network Structure

**Figure 5:**
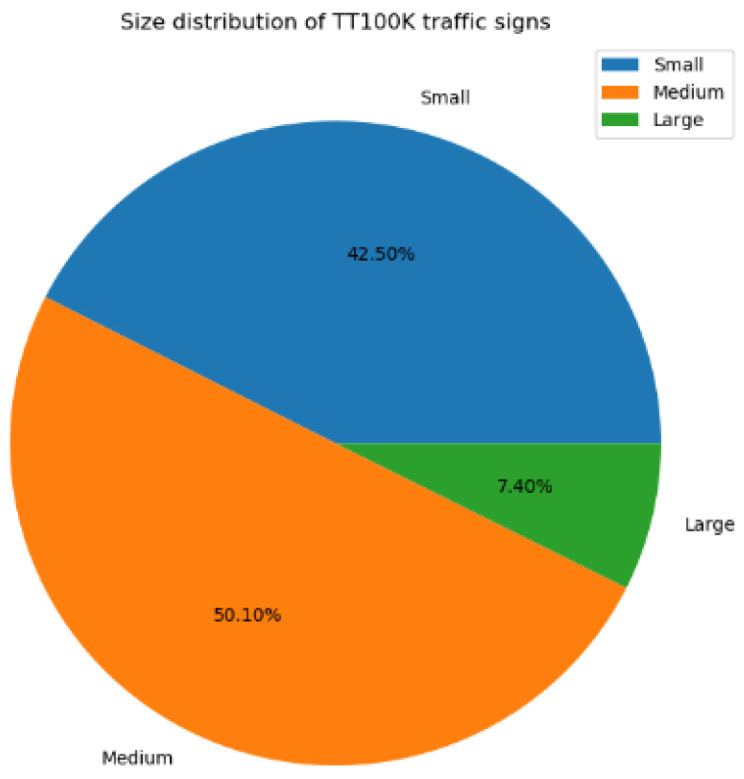
Size distribution of TT100K traffic signs.

The basic feature extraction network is commonly downsampled five times, with a downsampling rate of 2, and the multiplicity of five times downsampling is 32 to the fifth power of two, according to the COCO dataset description. If downsampling is continued, the feature map obtained will be one, and the target information will be lost. Small targets are fewer than 32 × 32 pixels, medium targets are 32 × 32–96 × 96 pixels, and giant targets are greater than 96 × 96 pixels [22]. As illustrated in Figure 5, the TT100K traffic sign dataset used in this work was mostly made up of small and medium targets, with large targets accounting for just 7.4% of the total dataset and tiny targets accounting for 42.5% [23].

The TT100K dataset has a high resolution, with each image having a resolution of 2048 × 2048 pixels and the largest traffic signs among the small targets accounting for less than 0.1% of the entire image, posing a significant challenge to the target detection algorithm. Small targets have limited features and necessitate great localization precision. Despite the introduction of the FPN structure in YOLOv3 to leverage multi-scale feature fusion to produce predictions by fusing the findings of distinct feature layers, which is critical for small target identification, the results were still unsatisfactory.

In the YOLOv3 network, the shallow layer contains less feature semantic information but a precise target location, whereas the deep layer has more but a coarse target location. As a result, shallow convolutional layers are used to predict small targets, and deep convolutional layers are used to predict large targets. A fourth feature prediction scale of size 152 × 152 was added to the three feature prediction scales of the YOLOv3 network structure in order to fully utilize the shallow features in the network to anticipate small targets. With an input image size of 608 × 608, the output image feature size was 152 × 152 after convolution and a two-fold upsampling of the input image, and the feature layer was induced through the route layer; this feature extraction was fused with the 11th layer feature to increase the fourth feature prediction scale.

**Figure 6:**
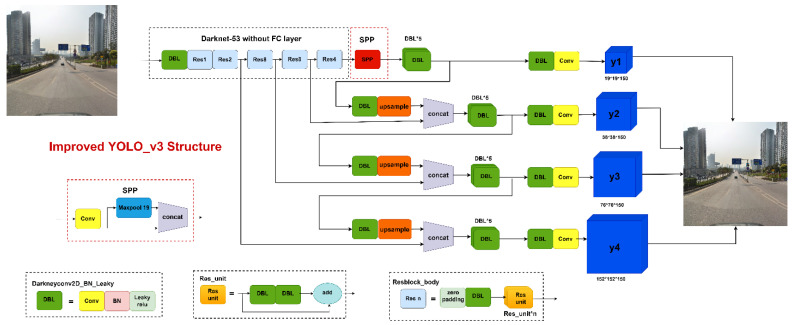
Structure of the improved YOLOv3 network.

In addition, the SPP module was added to realize the merging of local and global features by borrowing the notion of SPPNet and combining it with YOLOv3. Before the YOLO detection layer, the SPP module was integrated between the fifth and sixth convolutional layers, and the SPP module’s feature maps and feature maps pooled were reconnected and passed to the next detection network layer. To accomplish the feature map level fusion of local and global features, the SPP module’s maximum pooling kernel should be as close to the size of the feature map to be pooled as possible. To minimize the computational effort caused by the SPP module, enrich the feature map expression capability, and increase the detection impact, the SPP module in this research was composed of two parallel branches, each of which was composed of a 19 × 19 max pooling layer and a jump connection. Figure 6 depicts the improved YOLOv3 network structure.

### 3.2. Improved Loss Function

The loss function of YOLOv3 is composed of the center coordinate loss (*loss_xy_*), width–height coordinate loss (*loss_wh_*), confidence loss (*loss_conf_*), and classification loss (*loss_cls_*).

The central coordinate loss is represented by:

(1) $${loss}_{xy}=\lambda_{\mathrm{coord}}\overset{S^{2}}{\underset{i=0}{\sum }}\overset{B}{\underset{j=0}{\sum }}I_{ij}^{\mathrm{obj}}\left[{\left(x_{i}-{\hat{x}}_{i}^{j}\right)}^{2}+{\left(y_{i}-{\hat{y}}_{i}^{j}\right)}^{2}\right]$$

loss of width and height coordinates is represented by*:*

(2) $$\;{\;loss}_{wh}=\lambda_{\mathrm{coord}}\overset{S^{2}}{\underset{i=0}{\sum }}\overset{B}{\underset{j=0}{\sum }}I_{ij}^{\mathrm{obj}}\left[{\left(\sqrt{w_{i}^{j}}-\sqrt{{\hat{w}}_{i}^{j}}\right)}^{2}+{\left(\sqrt{h_{i}^{j}}-\sqrt{{\hat{h}}_{i}^{j}}\right)}^{2}\right]$$

confidence loss is represented by:

(3) $$\begin{array}{l} {\;loss}_{conf}=\overset{S^{2}}{\underset{i=0}{\sum }}\overset{B}{\underset{j=0}{\sum }}I_{ij}^{\mathrm{obbj}}\left[{\hat{C}}_{i}^{j}\log\left(C_{i}^{j}\right)+\left(1-{\hat{C}}_{i}^{j}\right)\log\left(1-C_{i}^{j}\right)\right]\\ -\lambda_{\mathrm{noobj}}\overset{S^{2}}{\underset{i=0}{\sum }}\overset{B}{\underset{j=0}{\sum }}I_{ij}^{\mathrm{noobj}}\left[{\hat{C}}_{i}^{j}\log\left(C_{i}^{j}\right)+\left(1-{\hat{C}}_{i}^{j}\right)\log\left(1-C_{i}^{j}\right)\right] \end{array}$$

and category loss is represented by:

(4) $${loss}_{cls}=-\overset{S^{2}}{\underset{i=0}{\sum }}I_{ij}^{\mathrm{obj}}\underset{c\in \mathrm{classes}}{\sum }\left[{\hat{P}}_{i}^{j}\log\left(P_{i}^{j}\right)+\left(1-{\hat{P}}_{i}^{j}\right)\log\left(1-P_{i}^{j}\right)\right]$$

where $\lambda_{\mathrm{coord}}$ denotes the coordinate loss weight; $\lambda_{\mathrm{noobj}}$ denotes the confidence loss weight without an object; $I_{ij}^{\mathrm{obbj}}$ denotes whether the *j*th anchor box of the *i*th cell is responsible for the object (1 or 0); $I_{ij}^{\mathrm{nobbj}}$ denotes the *j*th anchor box of the *i*th grid that is not responsible for the object; ($x_{i}$,$y_{i}$,$w_{i}^{j}$,$h_{i}^{j}$,$C_{i}^{j}$,$P_{i}^{j}$) denotes the predicted target box coordinates, confidence, and category; and (${\hat{x}}_{i}^{j}$,${\hat{y}}_{i}^{j}$,${\hat{w}}_{i}^{j}$,${\hat{h}}_{i}^{j}$,${\hat{C}}_{i}^{j}$,${\hat{P}}_{i}^{j}$) denotes the real target box coordinates, confidence, and category.

The YOLOv3 loss function is represented by Equation (5), where the mean square error (MSE) loss function is used for the bounding box regression and cross entropy is utilized as the loss function in *loss_conf_* and *loss_cls_*.

(5) $${loss=loss}_{xy}+{loss}_{wh}-{loss}_{conf}-{loss}_{cls}$$

However, utilizing MSE as the bounding box regression’s loss function is unfavorable to small target detection, sensitive to object scale, and focuses on big-scale targets while being unfriendly to small-scale objects. To balance the loss of large and small targets and maximize the detection results by weakening the influence of the bounding box size on the width and height loss function, the IoU-type loss function was employed in this paper, and the metric loss generated by IoU was used as a performance Equation (6).

(6) $$IoU=\frac{\left|A\cap B\right|}{\left|A\cup B\right|}$$

When the bounding box and the target box do not overlap, IoU = 0 does not reflect the distance gap between the two boxes; when the prediction box and the labeled box completely overlap, IoU = 1, the bounding box’s center point cannot be determined, and the size gap with the target box cannot be further optimized. DIoU loss [24] is independent of size; thus, big sizes will not result in a large loss. Due to the fact that a tiny size produces a little loss, which can address the problem, this work used the DIoU loss, whose calculation formula is presented in Equation (7).

(7) $$DIoU\;loss=1-IoU+\frac{\rho^{2}\left(b,b^{gt}\right)}{c^{2}}$$

where **b** and **b*^gt^*** denote the central points, *ρ* is the Euclidean distance, and *c* is the diagonal length of the smallest enclosing box covering the two boxes.

DIoU loss minimizes the distance between two target frames directly, converges quickly, and is more in line with the target frame regression mechanism, which takes into account the distance between the target and anchor, the overlap rate, and the scale, making target frame regression more stable, while still providing the gradient direction for the bounding box when it does not overlap with the target frame.

### 3.3. Generating Priori Frames Based on K-Means Clustering Algorithm

**Figure 7:**
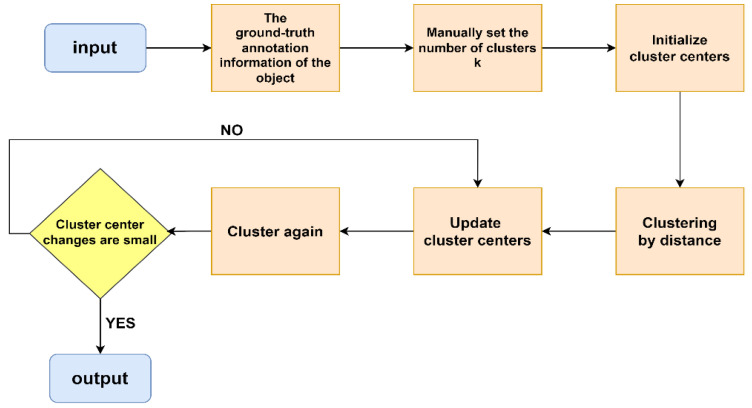
K-means clustering algorithm flow chart.

The anchor mechanism was implemented in YOLOv2, and the number of anchors was increased to nine in YOLOv3 to make the generated candidate regions more similar to the genuine labeled frames and boost the detection network’s recall. It was not appropriate to use the original anchor, since traffic signs are primarily small and medium targets, with fewer large targets in the TT100K dataset. For a specific dataset, choosing a suitable initial anchor can improve the detection effect, make the network easier to learn, and increase the detection rate of the bounding box. The flow of the K-means clustering algorithm to obtain candidate boxes is shown in Figure 7. In the TT100K dataset, the enhanced YOLOv3 network structure included a feature prediction scale, resulting in four scales and twelve anchors: (4, 5), (5, 6), (7, 7), (7, 13), (8, 8), (9, 10), (11, 12), (13, 14), (16, 17), (20, 22), (27, 29), and (41, 44).

## 4. Experiments and Analysis of Results

### 4.1. Dataset and Evaluation Indicators

**Figure 8:**
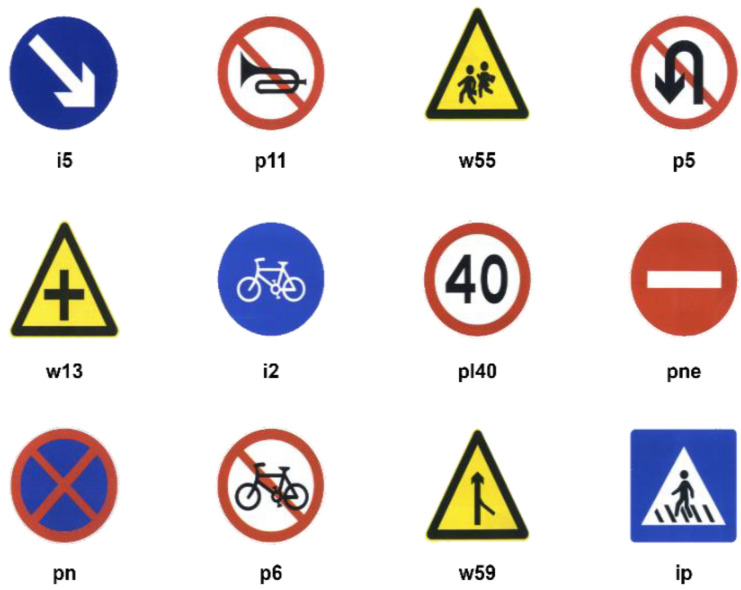
TT100K dataset partial traffic signs and category information.

There are a few big, publicly available traffic sign datasets, the majority of which use the GTSDB, but the GTSDB is not the same as Chinese traffic signs. CTSDB, CCTSDB, and TT100K, among others, are Chinese traffic sign datasets. The CCTSDB was expanded on the basis of CTSDB, and its categories were divided into warning signs, directional signs, and prohibition signs, without detailed classification of traffic signs. The TT100K traffic sign collection was created in collaboration between Tencent and Tsinghua University. It offered thorough categorization and identification of traffic signs, covered various climatic and lighting circumstances, and was more accurate for actual driving situations. Therefore, TT100K traffic sign dataset was used in this paper, and some of the traffic signs and the category information are shown in Figure 8.

**Figure 9:**
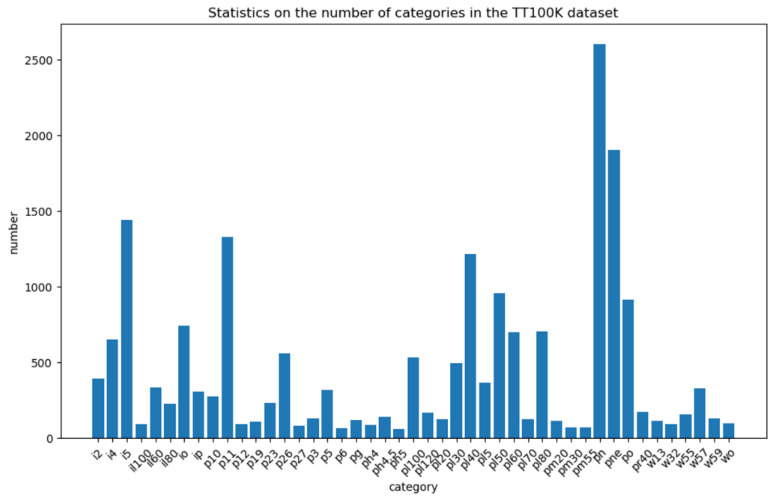
TT100K-45 comparison of the number of each traffic sign category.

The TT100K dataset has 100,000 photos with a resolution of 2048 × 2048 pixels, although there are unlabeled traffic sign images, and some categories have only a few images or duplicate images, reducing the detection effect. Therefore, this paper removed the unlabeled and duplicate traffic sign images from the dataset and selected 45 categories with a high number of traffic signs, where the 45 traffic sign categories were: pn, pne, i5, pl1, pl40, po, pl50, pl80, io, pl60, p26, i4, pll00, pl30, il60, pl5, i2, w57, p5, p10, ip, pl120, il80, p23, pr40, ph4. 5, w59, p12, p3, w55. pm20, pl20, pg, pl70, pm55, il100, p27, w13, p19, ph4, ph5, wo, p6, pm30, and w32, and the number of each traffic sign category is shown in Figure 9.

**Figure 10:**
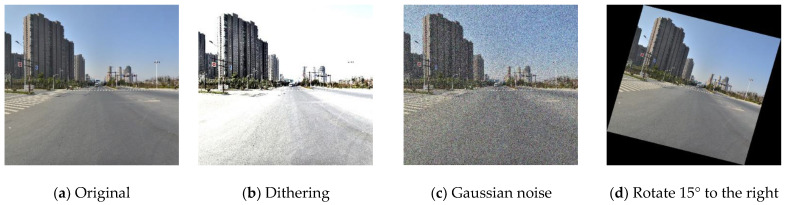
Strategies for dataset balancing and expansion.

Figure 9 shows that even if 45 categories with a large number of traffic signs were chosen, there was still a significant imbalance in the amount of data between each category, resulting in poor model prediction accuracy. As a result, as illustrated in Figure 10, this work balanced and expanded the dataset by employing tactics such as color dithering, Gaussian noise, and image rotation to ensure that the amount of each category was as equal as feasible.

The Mosaic approach reads four images at a time, scales and alters the color gamut of each image, arranges them in four directions, and then stitches the images together to create the target’s true frame. The enhancement method stitches four images, which is equivalent to calculating the parameters of four images with one input. This can reduce the number of images for batch input, reduce the training difficulty and training cost, improve the training speed, and largely enrich the number of samples in the dataset, which is conducive to the learning of features by the model.

In this paper, the evaluation metrics of the COCO dataset, including mAP_IoU = 0.50_, AP_S_, AP_M_, AP_L_, and several other metrics, were used to evaluate the performance of the model. In particular, most of the traffic signs in the TT100K traffic sign dataset belonged to small targets, so special attention needed to be paid to the detection accuracy of small targets. The specific meanings of the evaluation metrics are as follows:

AP: The area below the P–R curve, where P–R is precision and recall, respectively.

mAP_IoU = 0.50_: When the IoU threshold is set to 0.50, it is the average of all categories of AP in the dataset, which is the evaluation index of the PASCAL VOC dataset and corresponds to AP_IoU = 0.50_ in the COCO evaluation index.

AP_S_: average value of mAP for small objects: area < 322, and IoU = range (0.5, 1.00, 0.05) for a total of 10 IoUs.

AP_M_: medium objects: 322 < area < 962, and IoU = range (0.5, 1.00, 0.05) mean value of mAP for a total of 10 IoUs.

AP_L_: average value of mAP for large objects: area > 962, and IoU = range (0.5, 1.00, 0.05) for a total of 10 IoUs.

### 4.2. Experimental Results and Analysis

#### 4.2.1. Improved YOLOv3 Comparison Experiment

**Figure 11:**
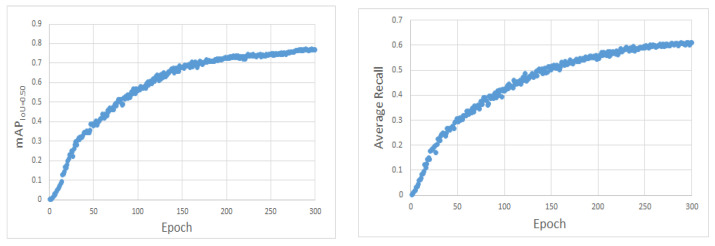
M-YOLOv3 mAP and AR trained on the TT100 dataset.

**Figure 12:**
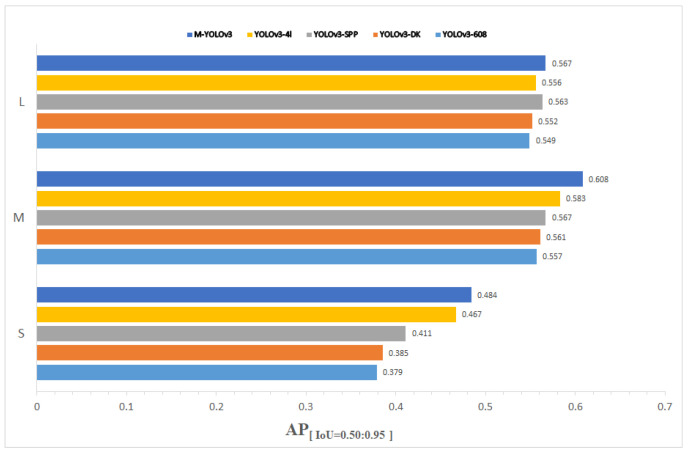
Plot of M-YOLOv3 versus other improved strategies on the TT100K dataset.

Three YOLOv3 networks with enhanced methods were compared and tested in this study, utilizing the TT100K traffic sign dataset and input images that were 608 × 608 pixels in size. Figure 11 displays the mAP and AR of M-YOLOv3 trained on the TT100 dataset. The detection results for various sizes of targets are shown in Figure 12 and Table 1. Among them, YOLOv3-DK adopted the strategy of improving the loss function DIoU loss and the re-clustering anchor; YOLOv3-SPP adopted the fusion space strategy of the pyramid pooling structure; YOLOv3-4l adopted the strategy of adding the fourth prediction feature layer with 152 × 152 scales; and M-YOLOv3 was the YOLOv3 network structure using all the improved strategies.

**Figure 13:**
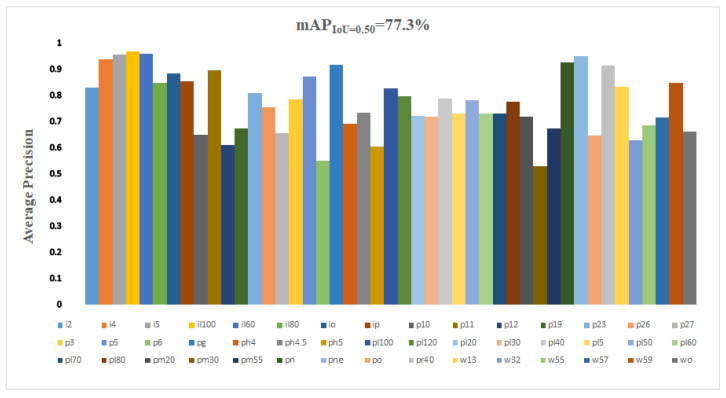
Test results of M-YOLOv3 on TT100K.

Table 1 and Figure 12 show that the average mean accuracy of the original YOLOv3 without employing any strategies was 68.9%, whereas the mAP of the upgraded YOLOv3 with all methods was 77.3%, an improvement of 8.4% in detection. The DIoU loss function and re-clustering anchor technique enhanced detection accuracy by 1.3%; however, the improvement was due to faster loss function convergence during training, which made the target box regression more stable and improved the recall rate. More pronounced improvements in mAP were seen in YOLOv3, which included an SPP structure and achieved a 73.2%. The SPP structure combined local and global characteristics, enhancing the feature map’s ability to express itself and significantly increasing detection accuracy. Using the method of adding a fourth prediction feature layer with 152 × 152 scales, the mAP was also considerably improved. The accuracy of tiny-target detection was enhanced by 10.5% when compared to YOLOv3, which made full use of the shallow features in the network for small-target prediction, resulting in a considerably improved detection effect, but at the cost of increased network complexity and processing. The best improvement was M-YOLOv3, which combined the three improvement procedures and achieved a mAP of 77.3%, which is 8.4% higher than the original YOLOv3′s average mean accuracy. Figure 13 depicts the test results of M-YOLOv3 on TT100K.

#### 4.2.2. Comparison of the Improved YOLOv3 Algorithm with Other Algorithms

M-YOLOv3 was compared with several other classical target detection algorithms to further validate the detection recognition of the improved network, and the results are shown in Table 2.

Table 2 demonstrates that M-YOLOv3 had the highest mAP of 77.3%, and SSD had the best real-time performance, with an FPS of 42. Compared with the original YOLOv3 algorithm, the average precision mean was greatly improved, although the real-time performance was reduced. Compared with the one-stage algorithm SSD, mAP improved by 12%, but there was still a gap in real-time performance. Compared with the two-stage target detection algorithm Faster-RCNN, the FPS was improved to 22, and the mAP was also improved by 1.7%, which improved the detection speed, as well as the detection accuracy. The trials showed that M-YOLOv3 performed better in terms of detection accuracy and speed.

#### 4.2.3. Improved Recognition Effect of YOLOv3 on Traffic Signs in a Special Environment

**Figure 14:**
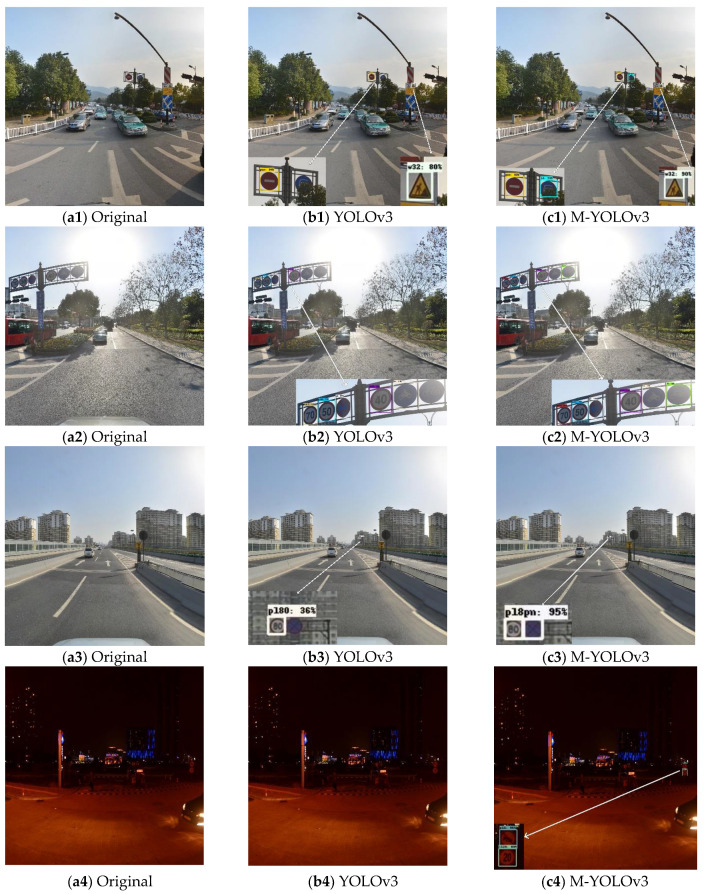
Comparison of the recognition effect of traffic signs under special environments.

Due to various factors, such as strong light irradiation, nighttime, and special environments of traffic sign occlusion, that will affect traffic sign detection and recognition in real-world driving scenarios, it was also necessary to consider the model’s recognition effect on traffic signs in special environments. In particular circumstances, the upgraded YOLOv3 model was employed to recognize traffic signs, as demonstrated in Figure 13. In Figure 14, the detection effect of YOLOv3 is compared with that of M-YOLOv3 in a special environment. As shown in Figure 14(b1,c1), the YOLOv3 algorithm failed to detect the obscured traffic sign in the case of an obscured traffic sign, while the improved YOLOv3 algorithm accurately identified the obscured traffic sign; as shown in Figure 14(b2,c2), the YOLOv3 algorithm had problems of false detection and missed detection for traffic sign recognition under the environment of strong light irradiation, while the improved YOLOv3 algorithm recognized all the traffic signs accurately. The improved YOLOv3 algorithm increased the fourth feature prediction scale for small targets, improving the detection effect of small targets, whereas the YOLOv3 algorithm had issues with missed detection and low confidence for small targets, as shown in Figure 14(b3,c3); in dimly illuminated environments, such as at night, the upgraded YOLOv3 algorithm recognized traffic signs, as illustrated in Figure 14(b4,c4); however the YOLOv3 method did not detect targets. As a result, under particular situations, the updated YOLOv3 algorithm still yielded better detection results.

## 5. Conclusions

A traffic sign detection and recognition network based on the modified YOLOv3 was suggested in this research, with the goal of addressing the difficulties of small targets being difficult to detect and low detection accuracy in traffic sign detection and identification tasks. The new spatial pyramidal pooling structure enabled the fusion of local and global features in this study, as well as increased the fourth feature prediction scale for small targets to improve the detection effect of small targets. To make the target frame regression more stable, the DIoU loss was utilized, which had a faster convergence and was more consistent with target frame regression. The detection network’s accuracy was considerably improved by damaging the real-time network as little as possible. The mAP increased by 8.4 points. The upgraded YOLOv3 algorithm enhanced the network’s complexity and lowered the detection speed. However, real-time detection is still a long way off; therefore, boosting detection speed to accomplish the effect of real-time detection will be the next research area.

## Figures and Tables

**Table 1:** Comparison of the improved YOLOv3 algorithm.

Algorithm	AP_S_	AP_M_	AP_L_	mAP_IoU = 0.5_
YOLOv3-608	0.379	0.557	0.549	0.689
YOLOv3-DK	0.385	0.561	0.552	0.702
YOLOv3-SPP	0.411	0.567	0.563	0.732
YOLOv3-4l	0.467	0.583	0.556	0.751
M-YOLOv3	0.484	0.608	0.567	0.773

**Table 2:** Comparison of improved YOLOv3 with other target detection algorithms.

Algorithm	mAP	FPS
YOLOv3	0.689	27
SSD	0.637	42
Faster-RCNN	0.756	2
M-YOLOv3	0.773	22

## Data Availability

Not applicable.
